# Seafloor Incubation Experiment with Deep-Sea Hydrothermal Vent Fluid Reveals Effect of Pressure and Lag Time on Autotrophic Microbial Communities

**DOI:** 10.1128/AEM.00078-21

**Published:** 2021-04-13

**Authors:** Caroline S. Fortunato, David A. Butterfield, Benjamin Larson, Noah Lawrence-Slavas, Christopher K. Algar, Lisa Zeigler Allen, James F. Holden, Giora Proskurowski, Emily Reddington, Lucy C. Stewart, Begüm D. Topçuoğlu, Joseph J. Vallino, Julie A. Huber

**Affiliations:** a Department of Biology, Widener University, Chester, Pennsylvania, USA; b Joint Institute for the Study of Atmosphere and Ocean, University of Washington, Seattle, Washington, USA; c NOAA/PMEL, Seattle, Washington, USA; d Department of Oceanography, Dalhousie University, Halifax, Nova Scotia, Canada; e Microbial and Environmental Genomics, J. Craig Venter Institute, La Jolla, California, USA; f Department of Microbiology, University of Massachusetts, Amherst, Massachusetts, USA; g MarqMetrix, Inc., Seattle, Washington, USA; h Great Pond Foundation, Edgartown, Massachusetts, USA; i Ecosystems Center, Marine Biological Laboratory, Woods Hole, Massachusetts, USA; j Marine Chemistry and Geochemistry, Woods Hole Oceanographic Institution, Woods Hole, Massachusetts, USA; Chinese Academy of Sciences

**Keywords:** RNA-SIP, autotrophy, deep sea, hydrothermal vent, instrumentation, metagenomics, metatranscriptomics

## Abstract

Depressurization and sample processing delays may impact the outcome of shipboard microbial incubations of samples collected from the deep sea. To address this knowledge gap, we developed a remotely operated vehicle (ROV)-powered incubator instrument to carry out and compare results from *in situ* and shipboard RNA stable isotope probing (RNA-SIP) experiments to identify the key chemolithoautotrophic microbes and metabolisms in diffuse, low-temperature venting fluids from Axial Seamount. All the incubations showed microbial uptake of labeled bicarbonate primarily by thermophilic autotrophic *Epsilonbacteraeota* that oxidized hydrogen coupled with nitrate reduction. However, the *in situ* seafloor incubations showed higher abundances of transcripts annotated for aerobic processes, suggesting that oxygen was lost from the hydrothermal fluid samples prior to shipboard analysis. Furthermore, transcripts for thermal stress proteins such as heat shock chaperones and proteases were significantly more abundant in the shipboard incubations, suggesting that depressurization induced thermal stress in the metabolically active microbes in these incubations. Together, the results indicate that while the autotrophic microbial communities in the shipboard and seafloor experiments behaved similarly, there were distinct differences that provide new insight into the activities of natural microbial assemblages under nearly native conditions in the ocean.

**IMPORTANCE** Diverse microbial communities drive biogeochemical cycles in Earth’s ocean, yet studying these organisms and processes is often limited by technological capabilities, especially in the deep ocean. In this study, we used a novel marine microbial incubator instrument capable of *in situ* experimentation to investigate microbial primary producers at deep-sea hydrothermal vents. We carried out identical stable isotope probing experiments coupled to RNA sequencing both on the seafloor and on the ship to examine thermophilic, microbial autotrophs in venting fluids from an active submarine volcano. Our results indicate that microbial communities were significantly impacted by the effects of depressurization and sample processing delays, with shipboard microbial communities being more stressed than seafloor incubations. Differences in metabolism were also apparent and are likely linked to the chemistry of the fluid at the beginning of the experiment. Microbial experimentation in the natural habitat provides new insights into understanding microbial activities in the ocean.

## Abstract (precis)

Diverse microbial communities drive biogeochemical cycles in Earth’s ocean, yet studying these organisms and processes is often limited by technological capabilities, especially in the deep ocean. In this study, we used a novel marine microbial incubator instrument capable of *in situ* experimentation to investigate microbial primary producers at deep-sea hydrothermal vents.

## INTRODUCTION

At deep-sea hydrothermal vents, low-temperature (i.e., diffuse) hydrothermal fluids emanating directly from igneous rock are hot spots of microbial primary production and provide access points to subseafloor habitats. Diffuse vents are formed when cold, oxidized seawater mixes with hot, chemically reduced hydrothermal fluids at and below the seafloor, creating steep geochemical gradients that support increased microbial biomass, activity, and diversity relative to the surrounding deep ocean (1–5). These fluids are dominated by chemolithoautotrophic bacteria and archaea that carry out a variety of metabolisms utilizing hydrogen, sulfur compounds, nitrate, and methane (6–12). However, our understanding of the impact of different microbial metabolisms on ocean biogeochemistry and the extent of carbon production from these reactions is nascent. This is partially due to the challenges associated with the collection and transfer of samples from the deep ocean to the surface for experimentation.

Samples transferred from the deep ocean to the sea surface are subject to changes in temperature and pressure and usually involve a long lag time between collection, sample recovery, and shipboard processing. Deep-sea devices designed for filtering seawater and other fluids at depth have been used to minimize these issues through *in situ* filtration, cell concentration, preservation, and analysis (reviewed in references 13 and 14). The outgassing of compounds such as hydrogen and carbon dioxide impacts microbial measurements from deep-sea hydrothermal vents; therefore, samples often need to be maintained at *in situ* pressures or temperatures when possible (reviewed in reference 15). For example, McNichol et al. (4, 16) used an isobaric gastight fluid sampler to conduct shipboard carbon fixation experiments with diffuse vent fluids maintained at *in situ* pressures and temperatures. However, these and other such experiments have sample processing delays and lack *in situ* preservation. This could be critical when sampling microbial communities in diffuse fluids that are in an extreme state of chemical disequilibrium and will likely undergo redox reactions between sampling and arrival in shipboard laboratories, regardless of the temperature and pressure conditions maintained in the sampling device.

A limited suite of samplers has been developed to carry out experiments while deployed in the ocean, keeping the instrument submerged for the duration of the experiment and fixing the samples post-experiment, before instrument recovery. This avoids biases related to sample collection lag and depressurization, although other experimental artifacts such as bottle effects remain (reviewed in references 14 and 17). One such instrument is the automated microlaboratory designed to conduct multiple (in-series) tracer incubation studies during cabled or free-drifting deployments (18–21) as well as a modification of the instrument termed the microbial sampler-submersible incubation device (MS-SID), allowing *in situ* grazing incubation experiments together with *in situ* microbial sampling and preservation (13, 22, 23). Another instrument is the environmental sample processor (ESP) unit, which includes a molecular component that carries out sample homogenization and the subsequent detection of particular microbial groups using quantitative PCR, sandwich hybridization, or a competitive enzyme-linked immunosorbent assay (ELISA) (24). A version of the ESP has successfully been deployed in the deep ocean, including in venting hydrothermal fluids (10) and methane seeps (25).

**FIG 1:**
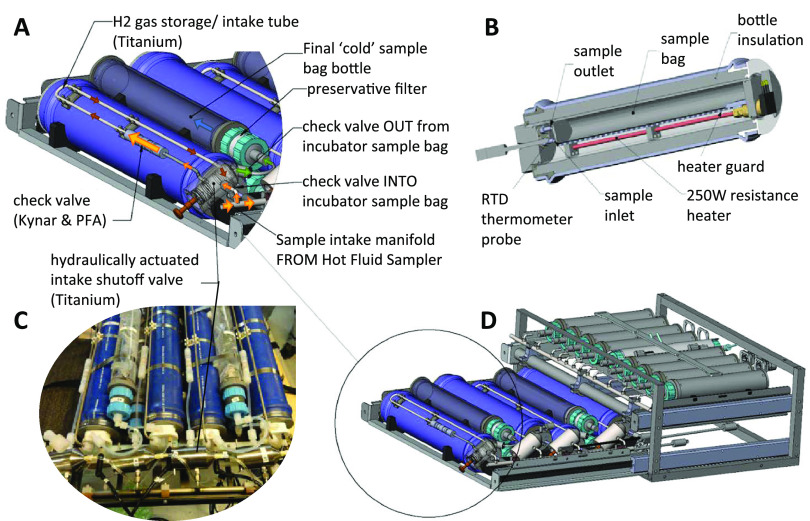
Incubator setup for the *in situ* RNA stable isotope probing (RNA-SIP) experiments. Each of the four incubation chambers was heated to a chosen set point temperature. Fluid was pulled into the insulated incubation chamber from the manifold of the hydrothermal fluid and particle sampler (HFPS) through a custom titanium shutoff valve, pulling hydrogen gas and buffering acid into the chamber as it filled. (A) After the incubation period, the fluid was pulled from the incubation chamber through a 0.22-μm filter with the passive addition of an RNA preservative. (B) A cutaway view of the incubation chamber shows the incubation bag over the heating element, with the RTD used to monitor the chamber temperature near the end of the bag. (C and D) The fully assembled incubator module (as deployed in 2015) (C) slides into the HFPS sample rack (D). Fluid transfer is accomplished with the HFPS sample pump and selection valve. PFA, perfluoroalkoxy.

We recently developed a shipboard RNA stable isotope probing (RNA-SIP) procedure combined with metatranscriptomics to identify the active chemolithoautotrophs and metabolic processes being performed during the uptake of dissolved inorganic carbon (DIC) at deep-sea hydrothermal vent ecosystems (6, 12). In the present study, the method was extended to the seafloor by running RNA-SIP experiments in a newly developed incubator that collects, heats, incubates, manipulates, and preserves seawater and vent fluids to allow *in situ* experimentation while being powered by a remotely operated vehicle (ROV) (Fig. 1). The major advantage of using the seafloor incubator is the ability to complete an entire experiment at specific temperatures immediately after sample collection while also controlling for both pressure changes and sample processing delays. As such, the results of the *in situ* incubation experiment were compared with those of parallel shipboard experiments in order to determine the effect of pressure changes and lag time on microbial metabolism. Here, we describe the new *in situ* incubator and the results of metatranscriptomic sequencing of communities from the shipboard and seafloor RNA-SIP experiments to provide new insights into the activities of natural microbial assemblages under nearly native conditions in the deep ocean.

## RESULTS

### ^13^C enrichment observed in RNA-SIP experiments.

Diffuse hydrothermal fluid at marker 33 vents directly from cracks in basalt along the eruption zone on the southeast side of the Axial caldera. Chemical analysis of this fluid is shown in Table S1 in the supplemental material. The fluid was 85% seawater and 15% hydrothermal end-member fluid based on the magnesium concentration (7). The temperature was monitored throughout the experiment, and temperature records showed that the incubator (Fig. 1) rapidly heated the chambers to 55°C and maintained the temperature within 2°C for the length of the incubations (Fig. S1). Upon recovery, the mass of each secondary bag was determined to indicate how much of the incubated sample was pulled through the RNA preservative filter at the end of the incubation. In general, the secondary bags were full or nearly full, and the primary incubator bags were empty or nearly empty. The pH of the filtered fluids in the secondary bags and from the shipboard incubation bottles was near 6 (Table S2).

**FIG 2:**
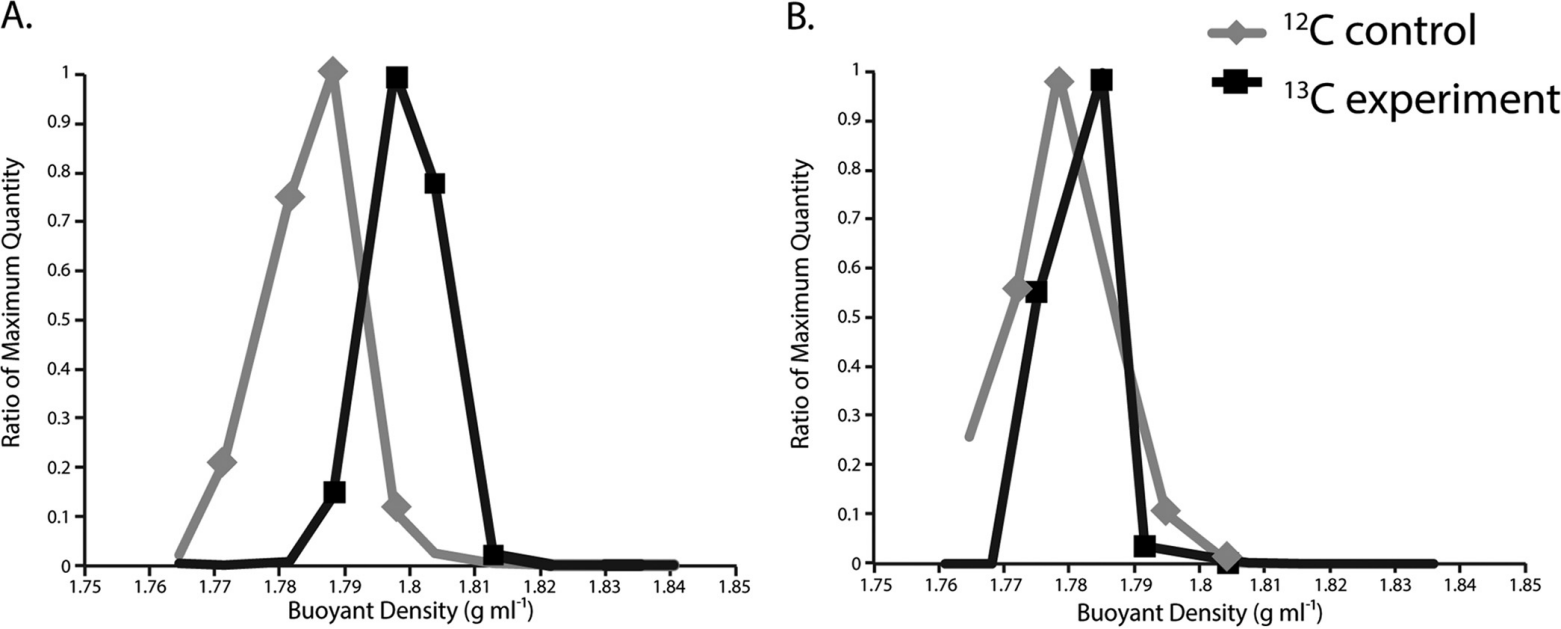
16S rRNA abundance in density gradient fractions of shipboard (A) and incubator (B) RNA-SIP experiments at 12 h. The buoyant density (grams per milliliter) of each fraction is depicted on the *x* axis, and the amount of 16S rRNA determined by RT-qPCR is on the *y* axis. The amount of 16S rRNA is displayed as the ratio of the maximum quantity in order to compare results between RNA-SIP experiments.

Both the 12-h and 16-h shipboard and incubator experiments showed ^13^C enrichment (Fig. 2; Fig. S2). Only the 12-h samples were sequenced to avoid heterotrophic cross-feeding from prolonged incubations. The maximum amount of 16S rRNA occurred at higher RNA densities in the ^13^C experiments than in the ^12^C controls (Fig. 2), indicating that dissolved inorganic carbon (bicarbonate) was incorporated into RNA during the incubations. For the shipboard experiment, the maximum amount of 16S rRNA occurred at densities of 1.788 g ml^−1^ and 1.804 g ml^−1^ for the ^12^C controls and the ^13^C experiment, respectively. For the incubator experiment, maximum 16S rRNA occurred at lower RNA densities overall, with peak amounts occurring at densities of 1.778 g ml^−1^ and 1.785 g ml^−1^ for the ^12^C controls and the ^13^C experiment, respectively (Fig. 2).

### Taxonomic composition of communities from the RNA-SIP experiments.

**FIG 3:**
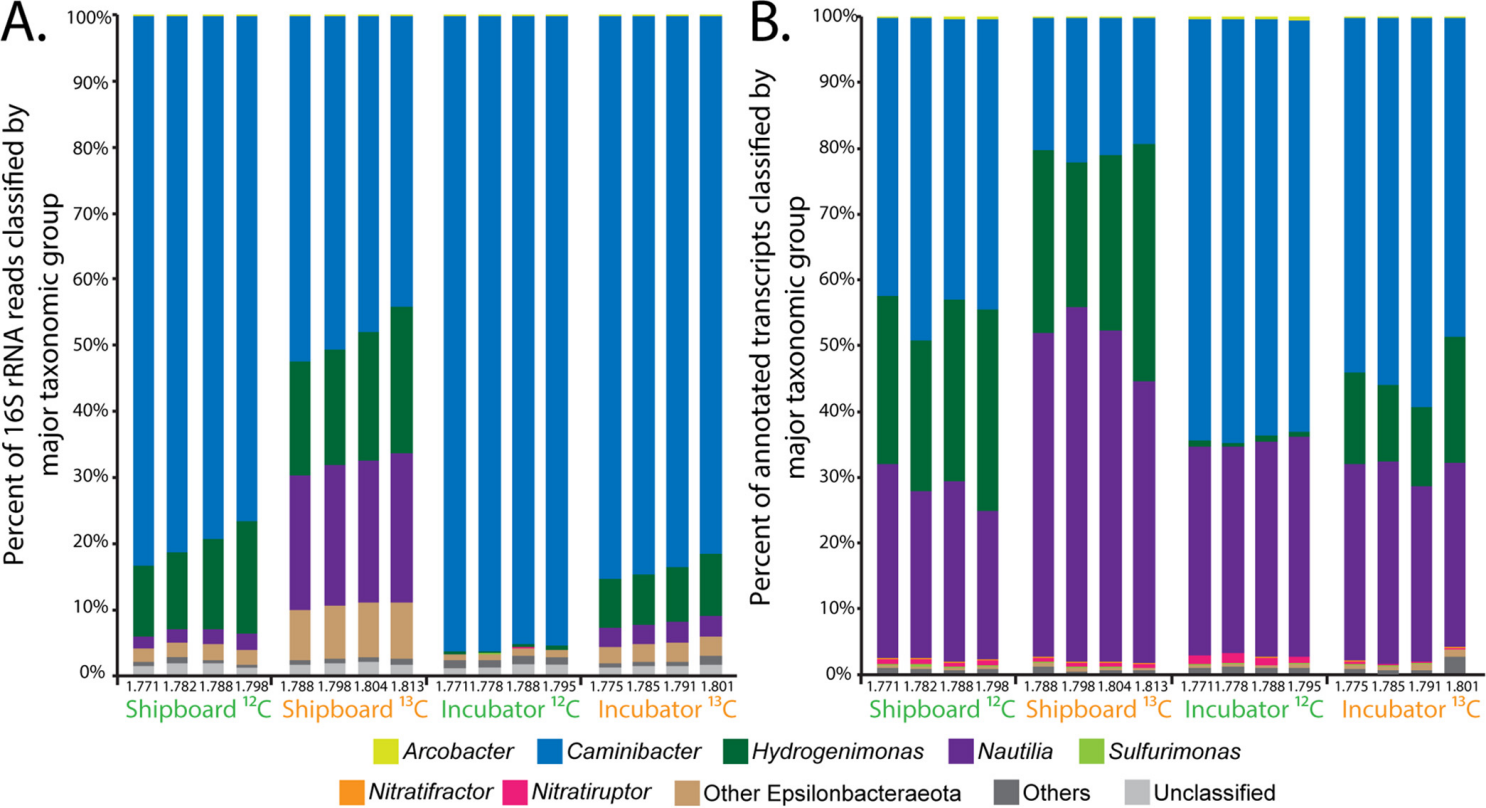
Taxonomic classification of 16S rRNA reads (A) and functionally (KO) annotated non-rRNA transcripts (B) from RNA-SIP metatranscriptomes.

The taxonomic composition of communities from the RNA-SIP experiments was determined based on the relative abundance of 16S rRNA sequences, and nearly all were primarily composed (96.7% to 98.2%) of thermophilic bacteria belonging to the *Epsilonbacteraeota* (Fig. 3A). The thermophilic genus *Caminibacter* was the most abundant in all SIP experiments, with relative abundances of 80.1%, 95.7%, and 83.7% for the ^12^C shipboard, ^12^C incubator, and ^13^C incubator metatranscriptomes, respectively (Fig. 3A). For the ^13^C shipboard experiment, *Caminibacter* was also the most abundant group but to a lesser extent, comprising 59.8% of the community, as this experiment also had higher relative abundances of both *Nautilia* and *Hydrogenimonas* 16S rRNA sequences (Fig. 3A). In the ^13^C shipboard experiment, *Nautilia* comprised 21.4% and *Hydrogenimonas* comprised 19.0% of the 16S rRNA sequences on average, indicating a different community composition in the ^13^C shipboard experiment than in the other experiments. *Hydrogenimonas* was more abundant in the shipboard community than in the incubator community, where it comprised only 0.4% of the ^12^C and 8.0% of the ^13^C incubator communities on average (Fig. 3A). This pattern was also observed in the taxonomic composition of the annotated transcripts (Fig. 3B). While *Caminibacter* comprised close to 50% of annotated transcripts in the ^12^C shipboard, ^12^C incubator, and ^13^C incubator metatranscriptomes, transcripts classified as *Nautilia* comprised a high percentage of the total annotated non-rRNA transcripts in all experiments (Fig. 2B).

**FIG 4:**
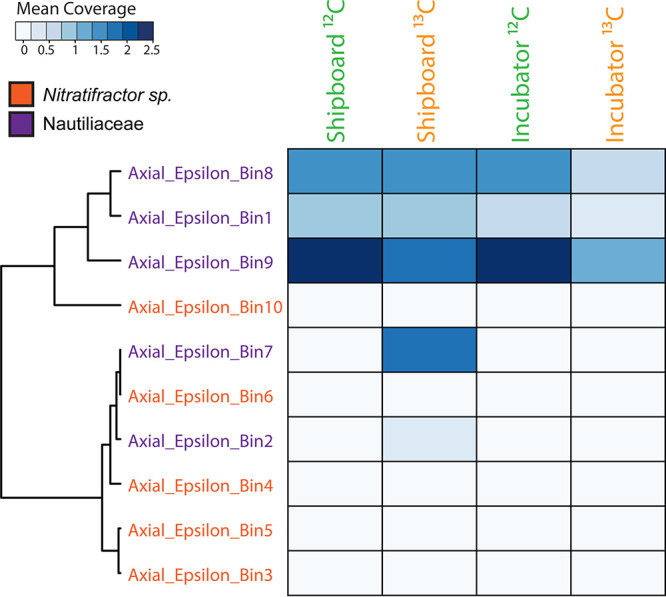
Heat map of mean coverage across the RNA-SIP experiments of metagenome-assembled genomes (MAGs) taxonomically identified as thermophilic *Epsilonbacteraeota*, specifically either the genus *Nitratifractor* or the family *Nautiliaceae*, as described previously (7). Fractions from each of the four RNA-SIP experiments have been collapsed, and mean coverage is summed. The scale depicts the range of mean coverages across MAGs. MAGs were clustered based on the similarity of coverage within the RNA-SIP experiments.

The mean coverage of metagenome-assembled genomes (MAGs) was used to determine the extent to which the MAGs were represented and active within the RNA-SIP experiments. Previously, 10 MAGs classified as thermophilic *Epsilonbacteraeota* (either *Nitratifractor* sp. or, more broadly, the family *Nautiliaceae*) were identified from marker 33 vent metagenomic assemblies as described previously (7). The 10 MAGs range from 13% to 94% completeness, as determined via Anvi’o. A heat map depicting the mean coverage of each MAG showed that these genomes were represented to various degrees in the 2015 marker 33 metagenome and were active in the marker 33 metatranscriptome as well as in the RNA-SIP metatranscriptomes (Fig. S3). Due to the higher coverage of the MAGs within the marker 33 metagenome, patterns among the SIP experiments were masked, and therefore, a second heat map was constructed showing only mean coverage across the SIP experiments (Fig. 4). The results showed that three MAGs (Axial Epsilon bins 1, 8, and 9) had the highest coverage across all SIP experiments. These three MAGs were broadly classified as belonging to the family *Nautiliaceae* (Fig. S7). The ^13^C shipboard experiment showed additional coverage of two other MAGs (Axial Epsilon bins 2 and 7), both also classified as belonging to the family *Nautiliaceae* (Fig. 4).

### Determination of metabolisms within RNA-SIP experiments.

Hierarchical clustering of all 16 RNA-SIP metatranscriptomes based on the normalized KEGG ontology (KO) abundance of annotated transcripts showed that the ^12^C controls for the incubator and shipboard experiments clustered together, indicating functional similarity between the shipboard and incubator SIP experiments (Fig. S4). The ^13^C experiments for the incubator and shipboard experiments clustered separately from the ^12^C controls and from each other, with the four ^13^C shipboard metatranscriptomes forming a separate cluster (Fig. S4).

When examining only the most abundant annotated transcripts expressed across all metatranscriptomes, the same clustering pattern was observed (Fig. S5). The most abundant transcripts were annotated to genes related to cell growth, translational processes, and energy metabolism. The gene to which the most annotated transcripts mapped was peroxiredoxin, a gene involved in reducing oxidative stress and, thus, cell damage. Other highly abundant transcripts were annotated to genes for elongation factors and molecular chaperones, indicating that translational machinery was active across all SIP experiments. In addition, transcripts for a key gene in the reductive tricarboxylic acid (rTCA) cycle, 2-oxoglutarate ferredoxin oxidoreductase, were also abundant, indicating that carbon fixation was occurring (Fig. S5). Additional transcripts for carbon fixation within the SIP experiments were also observed (Fig. S6). As observed in the taxonomic profiles, an examination of the most abundant annotated transcripts shows that the ^13^C shipboard metatranscriptome was slightly different from those of the other three experiments and clustered separately from the other metatranscriptomes (Fig. S5).

**FIG 5:**
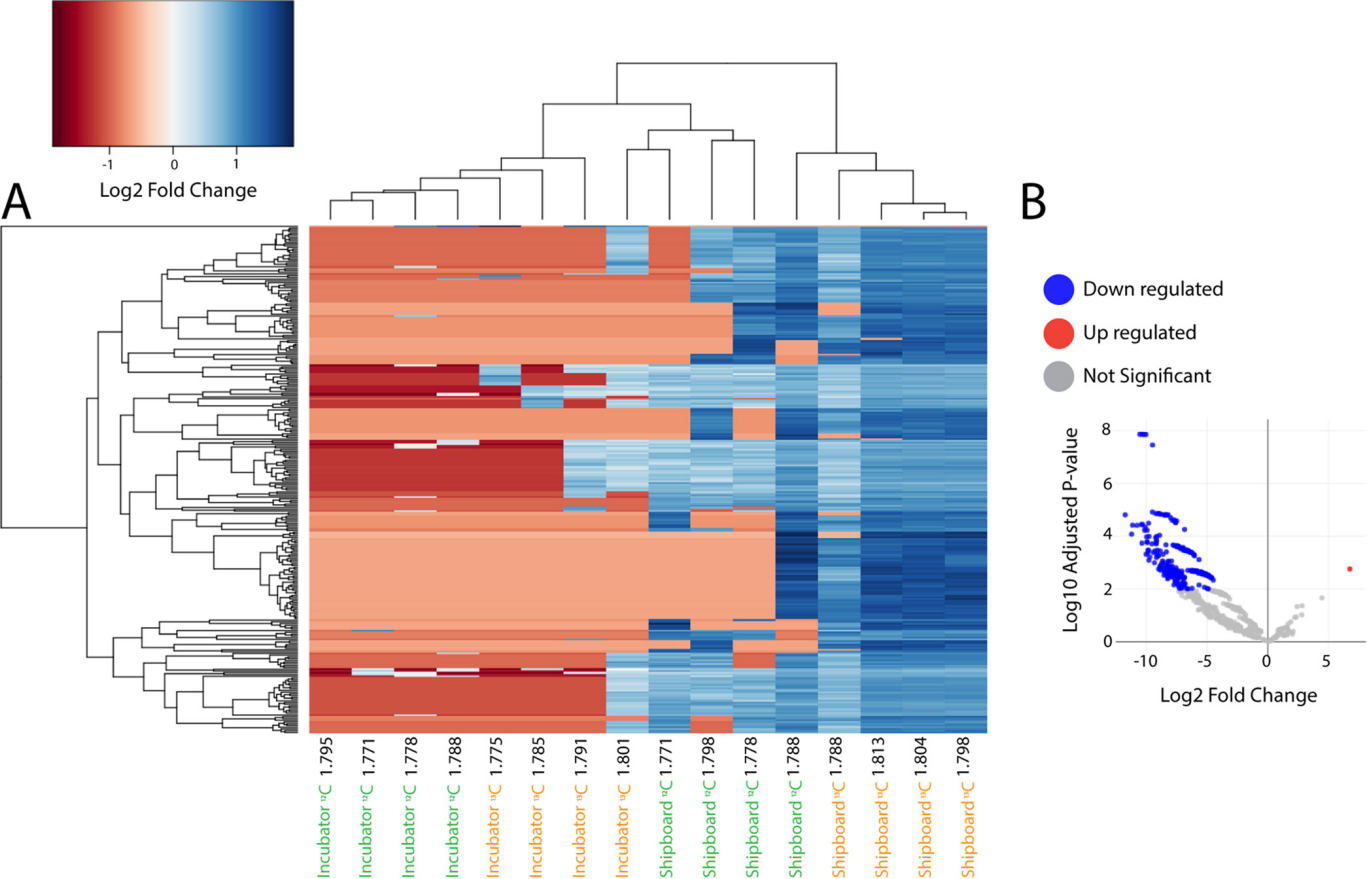
(A) Heat map showing the 233 KO annotated genes that were differentially expressed across fractions (adjusted *P* value of <0.01). (B) Volcano plot of the fold change in abundance versus the adjusted *P* value. Genes that were significantly upregulated (adjusted *P* value of <0.01) in the incubator versus shipboard fractions are in red, and downregulated genes are in blue.

Differential expression (DE) analysis was run to determine significant differences in annotated transcript abundances across the 16 RNA-SIP metatranscriptomes (Fig. 5). The results showed that 233 genes were significantly differentially expressed (adjusted *P* value of <0.01) in shipboard versus incubator RNA-SIP libraries, with all but 1 being more highly expressed in shipboard experiments than in incubator experiments (Fig. 5B). Annotated transcripts with the greatest differences in abundance (>10 log_2_ fold change) in shipboard versus incubator experiments included transcripts of genes related to translation, DNA replication, purine synthesis, and motility (Table S5).

**FIG 6:**
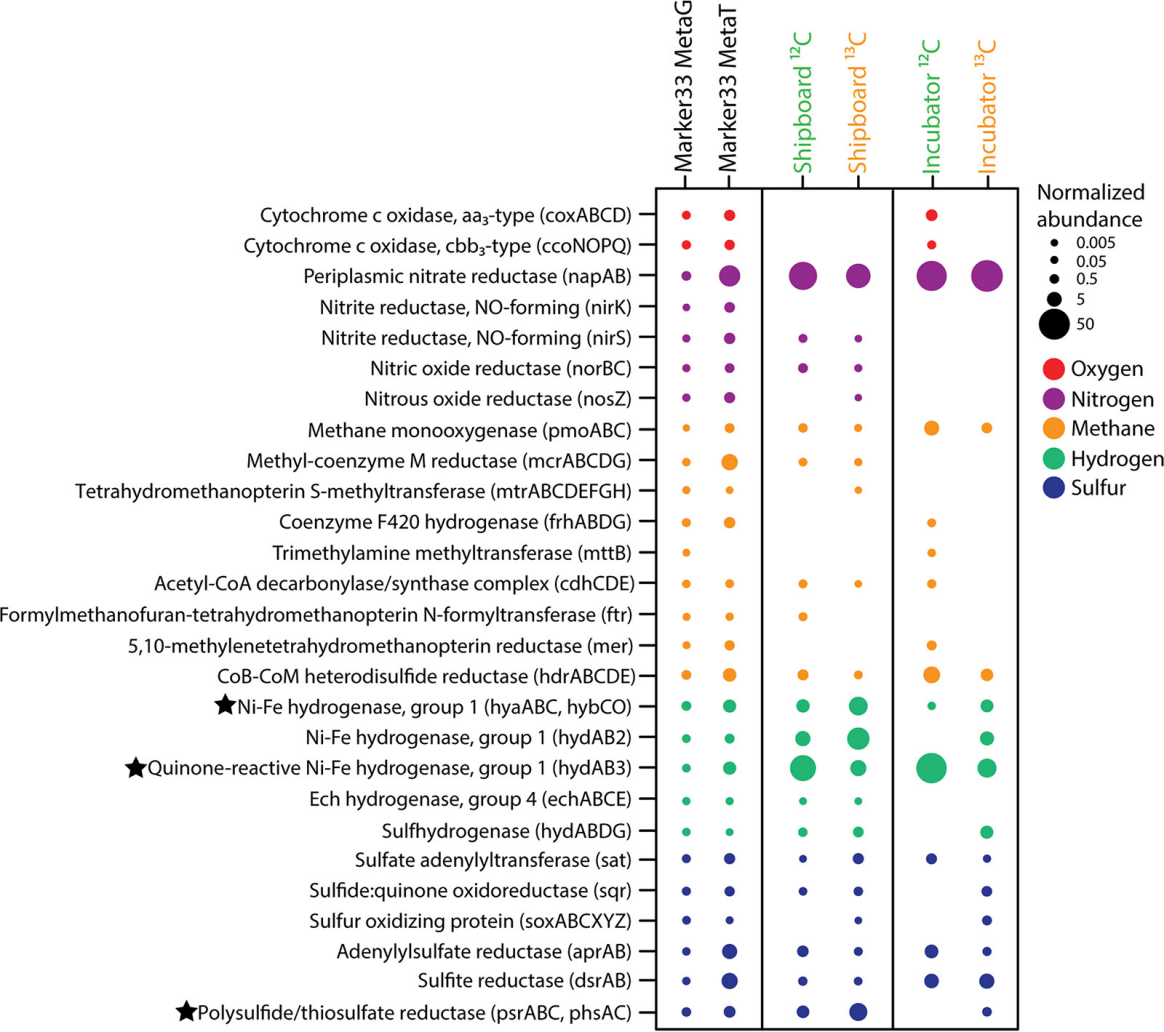
Normalized abundances of key genes and transcripts for oxygen, nitrogen, methane, hydrogen, and sulfur metabolisms within the 2015 marker 33 metagenome (MetaG) and metatranscriptome (MetaT) and the shipboard and incubator RNA-SIP experiments. Fractions from each of the four RNA-SIP experiments have been collapsed to reflect the normalized abundance of each gene in the entire experiment. Normalized abundances of metatranscriptomes were transformed to the same scale as the marker 33 metagenome. Black stars indicate a significant difference in transcript abundances (adjusted *P* value of <0.01) between shipboard and incubator RNA-SIP experiments. See Table S5 in the supplemental material for the specific subunits identified as significant.

The abundances of annotated transcripts involved in important metabolic processes differed between the shipboard and incubator SIP experiments (Fig. 6), although DE analysis revealed that many of these differences were not significant. The metatranscriptome of diffuse fluids reflects the diversity of metabolic processes that occur at a single vent, with the presence of annotated transcripts for aerobic respiration, denitrification, aerobic methane oxidation, methanogenesis, hydrogen oxidation, sulfur reduction, and sulfur oxidation. The reduced metabolic complexity observed within the RNA-SIP metatranscriptomes highlights the specific organisms and their metabolisms active under experimental conditions. Transcripts for the mainly anaerobic process of denitrification, specifically *nirS*, *norBC*, and *nosZ*, were observed only in the shipboard experiments. Conversely, transcripts for cytochrome *c* oxidases, important for aerobic respiration, were observed in only one of the incubator experiments (Fig. 6). Transcripts for methane metabolism differed, albeit not significantly, across experiments. Transcripts for the methyl-coenzyme M reductase gene (*mcrA*), important for anaerobic methanogenesis, were observed only in the shipboard SIP experiments. Aerobic methane oxidation transcripts, however, showed an average of a 1.35 log_2_ fold increase in incubator experiments compared to the shipboard experiments (Fig. 6). For hydrogen oxidation, transcripts annotated to genes for group 1 Ni-Fe hydrogenases (*hydA3* and *hyaC*) were more abundant in the shipboard experiments than in the incubator experiments (adjusted *P* value of <0.01). Sulfur metabolism transcripts for polysulfide and thiosulfate reduction showed significantly higher abundances (adjusted *P* value of <0.01) in the shipboard than in the incubator experiments (Fig. 6).

**FIG 7:**
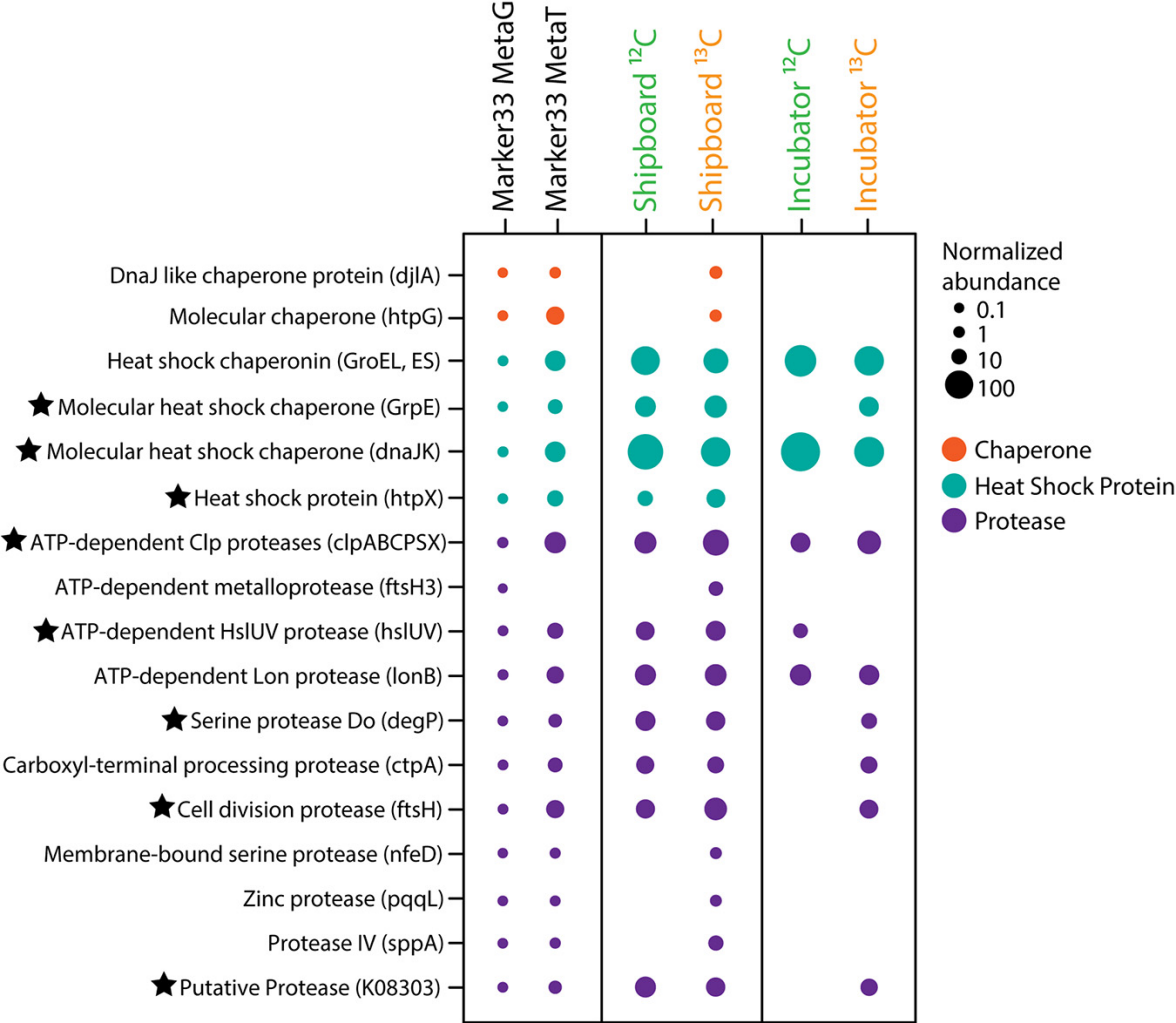
Normalized abundances of genes and transcripts annotated for cell stress, including genes for protein chaperones, heat shock proteins, and proteases, within the 2015 marker 33 metagenome and metatranscriptome and the shipboard and incubator RNA-SIP experiments. Fractions from each of the four RNA-SIP experiments have been collapsed to reflect the normalized abundance of each gene in the entire experiment. Normalized abundances of metatranscriptomes were transformed to the same scale as the marker 33 metagenome. Black stars indicate a significant difference in transcript abundances (adjusted *P* value of <0.01) between shipboard and incubator RNA-SIP experiments. See Table S5 in the supplemental material for the specific subunits identified as significant.

Because DE analysis indicated that the abundance of transcripts related to stress was significantly higher in the shipboard experiment than in the incubator experiment (Table S5), we further examined the abundance of transcripts related to stress (chaperones, proteases, and other heat shock proteins) within the samples (Fig. 7). An increase in the abundance of transcripts annotated to the heat shock chaperone genes *dnaK, dnaJ, groES*, and *groEL* was observed in both the shipboard and incubator experiments compared to the metatranscriptome of diffuse fluids (Fig. 7). However, *dnaJ* had a significantly higher abundance within the shipboard experiment (adjusted *P* value of <0.01). Additionally, transcripts for the heat shock protein gene *htpX* were expressed only in the shipboard SIP metatranscriptomes. Proteases, which play an important role in protein degradation during times of stress, were generally more highly expressed in the shipboard SIP experiments than in the incubator experiments. Specifically, transcripts for the protease genes *clpX*, *clpP*, *ftsH*, and *hslU* all showed significantly higher abundances in the shipboard experiments (adjusted *P* value of <0.01) (Fig. 7; Table S5).

## DISCUSSION

There are extreme technical challenges to understanding microbial life in the deep sea, and results and interpretations depend heavily on the experimental approach taken. Motivated by the hypothesis that chemical reactions and microbial activity that occur in sample containers between the time of sampling and the start of an experiment will affect experimental results significantly, we designed and built an *in situ* incubator to eliminate depressurization and lag time between sampling and experiments in order to better capture the *in situ* microbial activity at deep-sea hydrothermal vents where diffusely venting fluids exit the seafloor. We successfully demonstrated the ability to study thermophilic microbes close to their seafloor and subseafloor habitats and highlighted differences between shipboard and seafloor incubations. To probe these communities, an RNA-SIP methodology coupled to mRNA sequencing was applied to examine which organisms and metabolisms are responsible for autotrophy under experimental conditions that reflect those in the subseafloor (6). However, many different types of incubations at temperatures from ambient to at least 80°C are possible with this instrument, making it a valuable new tool for marine microbial ecology.

The marker 33 vent was chosen as the site for seafloor incubator testing due to the consistent presence of thermophilic bacteria and archaea detected in previous studies (7, 26–29). We chose RNA-SIP for the demonstration of the *in situ* autotrophic activity of subseafloor microbial communities, and these experiments must mimic the physical and chemical conditions of the environment as closely as possible, which is inherently challenging in any aquatic sample. For example, it may be hours before fluid collected on the seafloor can be dispensed into shipboard bottles and incubated, thus increasing the likelihood of changes to the microbial community. This time lag combined with pressure and temperature changes during the transport of diffuse fluids to the surface in unpressurized vessels may also result in outgassing of key redox species such as methane, hydrogen sulfide, hydrogen, and carbon dioxide (16, 30); the death of pressure- and temperature-sensitive organisms (31); or a loss of oxygen to microbial consumption or chemical reactions in the sample container (32). Performing experiments *in situ* on the seafloor may help ameliorate many of the biases introduced with shipboard experiments, but few direct comparisons between *in situ* and shipboard experiments exist.

Except for location (seafloor or shipboard) and timing after fluid sampling, all other conditions (temperature, pH, concentrations of hydrogen and DIC label added, and length of incubation) were identical between experiments. The pH of the incubations in both sets of experiments was similar to that of the vent fluid from marker 33. Both the shipboard and seafloor incubator experiments showed ^13^C enrichment relative to their ^12^C controls, with maximum 16S rRNA occurring at higher RNA densities. However, the RNA densities of the two experiments were slightly different, with peak 16S rRNA occurring at lower RNA densities overall in the incubator experiment. The reason for the lower level of enrichment in the seafloor incubator is unclear but may be due to differences in the dominant microbial genera present in each experiment or stochastic effects. The majority of the rRNA from all SIP experiments, both shipboard and incubator, was comprised of thermophilic *Epsilonbacteraeota* oxidizing hydrogen and reducing nitrate while fixing carbon, consistent with the native community present at the marker 33 site in 2015 as well as numerous omics surveys at diffuse vents, indicating that these organisms and metabolisms often dominate in the reducing, warm subseafloor habitat (4, 6, 7, 9, 10, 12, 33). There was a higher percentage of rRNA classified as belonging to the genera *Hydrogenimonas* and *Nautilia* in the shipboard experiments than in the incubator experiments (Fig. 3A), including two *Nautilia* populations observed only in the ^13^C shipboard experiment (Fig. 4). Based on publicly available genomes, *Nautilia* species have a higher GC content in their genomic DNA (average, 34.8%) than *Caminibacter* (average, 28.9%), which may account for the higher peak RNA density in the ^13^C shipboard than in the ^13^C incubator experiments.

Differences in metabolism were apparent between the shipboard and incubator experiments and may be linked to the chemistry of the fluid at the beginning of the experiment. For example, transcripts annotated for denitrification (*nirS*, *norB*, and *nosZ*) and methanogenesis (*mcrA*) were observed only in the shipboard experiments. Additionally, a significantly higher abundance of hydrogen oxidation transcripts (*hyaA3* and *hyaC* [adjusted *P* value of <0.01]) was observed in the shipboard than in the seafloor incubations. Although not significant, there was a higher abundance of transcripts annotated for aerobic methane oxidation (*pmoA*) and oxygen utilization (*cox* and *cco*) in the incubator experiments than in the shipboard experiments (Fig. 6). We hypothesize that during the lag time between sample collection and the beginning of the experiment shipboard (approximately 17.5 h), oxygen was consumed in the vent fluids by aerobic microorganisms and abiotic reactions with the high concentrations of dissolved sulfide and reduced metals in the samples. Therefore, by the time the fluid was used in the shipboard incubations, there was little to no oxygen left. For the seafloor experiment, incubations of samples that were approximately 85% deep seawater (see Table S1 in the supplemental material) contained oxygen at the start of the experiment, and oxygen-consuming microbes grew. Aerobic oxidations of methane and sulfur species are important microbial metabolisms in hydrothermal vent plumes as well as in many venting fluids where deep, oxygen-rich seawater mixes with the reducing vent fluids (34–36). For example, our metatranscriptomic study from multiple vent sites at Axial Seamount, including marker 33 in 2015, showed the transcription of cytochrome *c* oxidases and methane monooxygenase at this site, indicating that these processes were occurring *in situ* in the venting fluids (7). Additional *in situ* experiments focused on assessing the metatranscriptome of the incubated vent fluid over a shorter time scale might resolve an initial aerobic stage from a later anaerobic stage and capture some of the dynamic spatial variability in microbial activity around diffuse vent sites. Overall, these results highlight the importance of performing incubations *in situ* and demonstrate that incubations performed shipboard may underestimate aerobic metabolisms due to the consumption of oxygen during sample recovery.

In addition to differences in microbial metabolism, we found significantly higher abundances of transcripts annotated for heat shock proteins, proteases, and chaperones in the shipboard experiments than in the incubator experiments, which may indicate that the shipboard microbial community was under more thermal stress (37). Chaperones can aid in protein folding and prevent protein denaturation that occurs during environmental stress (37, 38). The abundance of transcripts for chaperone-encoding genes in both shipboard and incubator experiments was higher than for the marker 33 metatranscriptome (Fig. 7), an indication that experimental incubations, be it on the seafloor or shipboard, enact some stress on microbial communities. However, transcripts annotated for proteases and heat shock proteins were significantly more abundant in the shipboard experiments (adjusted *P* value of <0.01), particularly in the ^13^C experiment (Fig. 7). The increased environmental stress could be due to transport to atmospheric pressure, manipulation of fluid into glass bottles, or any number of differences that occur when carrying out incubations shipboard compared to incubating the fluid *in situ* on the seafloor. Another possibility is that the incubations were performed at temperatures near the optimal growth temperatures of *Caminibacter* (55°C to 60°C [39–41]), *Nautilia* (53°C to 60°C [42–44]), and *Hydrogenimonas* (55°C [45]), which may induce the transcription of thermal stress proteins in these organisms. Growth at pressures found at deep-sea vents increased the optimal growth temperature (46–48) and raised the thermal induction temperature (47) in hyperthermophilic archaea. Therefore, *in situ* incubation of vent fluids in this study may similarly ameliorate thermal stress in *Epsilonbacteraeota* relative to shipboard incubations.

In conclusion, this study showed the effects of depressurization and sample processing delays using a new *in situ* incubator instrument to carry out RNA-SIP experiments *in situ* on the seafloor. The taxonomic and functional gene differences observed between shipboard and incubator experiments were likely due to slight differences in the chemistry of the fluid at the start of the experiment and, more specifically, the availability of oxygen in the incubator experiment. Microbial populations were also more stressed in shipboard experiments. Although the shipboard and incubator experiments were similar, the slight differences between the two suggest that the use of a seafloor incubator may give a more accurate account of the microbial metabolic processes occurring within diffusely venting fluids due to reduced lag time, depressurization, and stress. In addition, the seafloor incubator limited both abiotic and biotic reactions that modify the chemistry of the fluids during transport to the ship.

The use of instrumentation like the seafloor incubator is an important step in understanding and constraining the roles that microbial communities play in the deep ocean, with potential applications well beyond those described here. The incubator can collect seawater, cold seep fluids, or vent fluids and their associated microbial communities and immediately amend the fluids while keeping them at *in situ* pressure and a controlled temperature before filtering and preserving the microbial biomass. Future experiments with the incubator will focus on performing quantitative time series measurements of microbial, viral, and geochemical activities for various biogeochemical processes as well as nutrient amendment experiments to measure the effect of substrate concentrations on reaction rates, chemical signatures, and microbial and viral community compositions and functions. Thus, our study expands our understanding of the activities of natural microbial assemblages under nearly native conditions at deep-sea hydrothermal vents and allows for future deployments to better constrain marine microbial biogeochemistry in the ocean.

## MATERIALS AND METHODS

### Fluid collection for omics.

Low-temperature (41°C) hydrothermal vent fluid was collected from the marker 33 vent at Axial Seamount, a submarine volcano located off the coast of Oregon (45.93346, −129.98225; 1,516-m depth) on 26 August 2015 on board the R/V *Thomas G. Thompson* using the ROV *Jason II*. Fluids were collected using the hydrothermal fluid and particle sampler (HFPS) (1), which has an integrated temperature sensor to continuously monitor fluid temperature during intake. The collection and processing of diffuse vent fluid samples for RNA-SIP are described below. For the collection of filtered vent fluid for microbial community DNA and RNA analyses, 3 liters of diffuse fluid was pumped through a 0.22-μm-pore-size, 47-mm-diameter filter (Millipore) and preserved immediately *in situ* with RNAlater to be used in metagenomic and metatranscriptomic library preparation as described previously (7). Separate fluid samples were collected and analyzed for alkalinity and hydrogen sulfide, ammonia, methane, and hydrogen concentrations according to methods described previously (1). The oxygen concentration and pH of the fluid were measured during intake using a Seabird 63 optical oxygen sensor and an AMT deep-sea glass pH electrode, which were integrated into the HFPS.

### Shipboard RNA stable isotope probing experiments.

Shipboard RNA-SIP experiments were performed as previously described (6). The HFPS was used to collect 4 liters of diffuse vent fluid into an acid-washed Tedlar bag. Approximately 17 h later, the vehicle was recovered, and ∼30 min later, diffuse fluid was pumped from the Tedlar bag into four evacuated 500-ml Pyrex bottles and filled to capacity (530 ml). Prior to filling, ^12^C-labeled sodium bicarbonate or [^13^C]sodium bicarbonate was added separately to a pair of bottles to reach a final added concentration of 10 mM bicarbonate. After adding the fluid sample to each bottle, 1 ml of 1.2 M HCl was added to counteract the added bicarbonate and ensure a pH similar to that of unamended vent fluid. H_2_ (900 μmol) was then added to each bottle. A pair of ^13^C- and ^12^C-labeled bottles was then incubated at 55°C for 12 h, while another pair was incubated for 16 h. After incubation, the fluid from each bottle was filtered separately through 0.22-μm-pore-size Sterivex filters, preserved in RNAlater, and frozen at −80°C.

### Seafloor incubator RNA stable isotope probing experiments.

The seafloor incubator units were incorporated as a module on the HFPS (Fig. 1) and designed to pull in vent fluid using the existing HFPS framework A/C powered by the submersible. The incubations occurred concurrently with other HFPS fluid collection and dive operations. The main components of a single incubator unit consisted of an insulated incubator bottle containing the primary sample bag (4-mil-thick Tedlar bag), an RTD (resistance temperature detector) probe, and a 250-W heating rod and a final bottle containing a secondary sample bag (2-mil-thick Tedlar bag) and a titanium shutoff valve situated between the fluid intake lines and the incubator bottle. Four insulated incubation units were loaded onto one rack of the HFPS (Fig. 1).

Prior to deployment, ^12^C-labeled sodium bicarbonate or [^13^C]sodium bicarbonate was added separately to a pair each of primary incubation bags to reach a final concentration of 10 mM added bicarbonate upon filling with 800 ml of vent fluid. The lines running to each bag were primed with 1.5 ml of 1.2 M HCl to ensure a pH similar to that of unamended vent fluid as well as 900 μmol of pure H_2_ to match the shipboard incubations. Approximately 1 h prior to fluid sampling on the seafloor, the insulated incubator chambers were heated to 55°C. This incubation temperature was selected based on the high abundances of thermophiles at marker 33 in previous studies (7, 26) and matched the incubation temperature of the shipboard RNA-SIP experiments described above. Once at temperature, the primary sample bags were filled with 800 ml of diffuse vent fluid using the HFPS as described above, and a shutoff valve was hydraulically closed to prevent further intake from the sample manifold. An RKC MA901 proportional-integral-derivative (PID) temperature controller housed in a separate titanium case recorded and controlled the incubator temperature from an RTD thermometer situated next to the bag and maintained a constant temperature at a set point (±2°C) by supplying variable power to the heating rod located beneath the Tedlar incubation bag inside the incubator (see Fig. S1 in the supplemental material). The PID control algorithm was tuned to the incubator bottle prior to deployment using the MA901 autotune feature. The heating rod induced convection in the incubation chamber, resulting in an even temperature distribution. The temperature distribution within the incubator sample bag was monitored during predeployment laboratory experiments and was found to vary less than 2°C (Table S4).

A pair of insulated incubator chambers containing ^12^C- and ^13^C-labeled bicarbonate was incubated for 12 h while an identical pair of chambers was incubated for 16 h. At the end of each incubation, fluid was pumped from the primary incubator bag through a 0.22-μm-pore-size polyethersulfone (PES) filter (Millipore) into a secondary bag that was surrounded by ambient seawater (∼2°C). Filters were preserved immediately *in situ* with RNAlater. Once shipboard, the fluids in the secondary sample bags were analyzed for pH, and the filters were frozen at −80°C.

### Fractionation for RNA-SIP experiments, RT-qPCR, library preparation, and analysis.

RNA from the incubator and shipboard SIP experiments was extracted, quantified, and fractionated after isopycnic centrifugation as described previously (6) and in the supplemental material. The 16S rRNA copy number was determined for each fraction via reverse transcription-quantitative PCR (RT-qPCR) with universal primers Pro341F and Pro805R (49), as described in the supplemental material. This measurement was used for comparison between the ^12^C and ^13^C samples and for the determination of ^13^C enrichment. Four fractions from each of the ^12^C and ^13^C samples from the shipboard and incubator samples were sequenced, including fractions with the maximum amount of 16S rRNA and a few fractions on either side of the peak, for a total of 16 metatranscriptomic libraries. RNA-SIP metatranscriptomic library preparation and downstream analyses were completed as described in detail in the supplemental material. Briefly, double-stranded cDNA was constructed from each RNA-SIP metatranscriptome and used for library preparation via the NuGen Ovation ultralow library DR multiplex system, according to the manufacturer’s instructions. rRNA was not removed prior to library construction. For the RNA-SIP metatranscriptomes, taxonomy, overall transcript abundance, and hierarchical clustering are displayed for all 16 libraries. For visualization of key metabolic processes, the 16 libraries were collapsed into their corresponding experiments: ^12^C shipboard, ^13^C shipboard, ^12^C incubator, and ^13^C incubator. The transcript abundance across fractions was summed for each experiment.

### Marker 33 metagenomic and metatranscriptomic library preparation and analysis.

The *in situ* preserved 47-mm-diameter flat filters were cut in half with a sterile razor, with each half being used for DNA and RNA extractions, respectively, and the corresponding libraries were prepared for sequencing as described previously (7) and in the supplemental material. Briefly, extracted RNA was treated using a Turbo-DNase kit (Ambion), purified, and concentrated using the RNeasy MinElute kit (Qiagen). rRNA removal, cDNA synthesis, and metatranscriptomic library preparation were carried out using the NuGen Ovation complete prokaryotic RNA-Seq dedicated read (DR) multiplex system according to the manufacturer’s instructions. Metagenomic library construction was completed using the NuGen Ovation ultralow library DR multiplex system according to the manufacturer’s instructions.

### Differential expression analysis.

Differential expression (DE) analysis was run to identify specific transcripts whose abundance was significantly higher or lower (adjusted *P* value of <0.01) between the shipboard and seafloor RNA-SIP experiments. DE analysis was run using the interactive tool DEBrowser in R (50). Within DEBrowser, differential expression analysis was run using normalized transcript abundances for KO annotated genes for all 16 RNA-SIP libraries with Limma, an R package used for the analysis of gene expression data (51). Low-count transcripts, defined as the maximum normalized abundance for each transcript across all samples being <10, were removed from the analysis. The resulting tables, heat maps, and plots showing significance were generated within DEBrowser.

### Mapping to thermophilic *Epsilonbacteraeota* MAGs.

Metagenome-assembled genomes (MAGs) were assembled and taxonomically identified from Axial Seamount metagenomic data as described previously (7). In this study, we determined the mean coverage of the 10 previously identified MAGs classified as thermophilic *Epsilonbacteraeota* via Phylosift (52) and CheckM (53) within the data set (Table S3). All reads from the marker 33 metagenome and marker 33 metatranscriptome and all RNA-SIP metatranscriptomes were mapped to each of the 10 MAGs using Bowtie2 with an end-to-end alignment and default parameters (v2.0.0-beta5) (54). The mean coverage for each MAG within the marker 33 metagenome was calculated via Anvi’o (55). The mean coverage for each MAG within the marker 33 metatranscriptome and RNA-SIP metatranscriptomes was calculated via samtools (56). For ease of visualization, the 16 RNA-SIP metatranscriptomes were collapsed, and the mean coverage for each MAG within fractions was averaged for each of the four experiments: ^12^C shipboard, ^13^C shipboard, ^12^C incubator, and ^13^C incubator. Heat maps of mean coverage were constructed in R using the package heatmap3 (v3.3.2) (57).

### Data availability.

Raw sequence data are publicly available through the European Nucleotide Archive (ENA), with project accession numbers PRJEB38697 for RNA-SIP metatranscriptomes and PRJEB19456 for the marker 33 metagenome and metatranscriptome. Assembled contigs for the marker 33 metagenome and RNA-SIP metatranscriptomes are publicly available via IMG/MER under submission numbers 78401, 78404, 97537, 97580, 97581, 97582, 97583, 97584, 97585, 97586, 97587, 97588, 97589, 97590, 97591, 97593, 97594, and 97595. Contigs for the 10 *Epsilonbacteraeota* MAGs are available through figshare at https://doi.org/10.6084/m9.figshare.5151547.v1.

## Supplementary Material

Supplemental file 1

Supplemental file 2
