# Supplementary material for: Seafloor Incubation Experiment with Deep-Sea Hydrothermal Vent Fluid Reveals Effect of Pressure and Lag Time on Autotrophic Microbial Communities
Source: Appl Environ Microbiol. 2021 Apr 13;87(9):e00078-21. doi: 10.1128/AEM.00078-21 (PMC8091007; doi:10.1128/AEM.00078-21)
Supplement: Supplemental file 1 [file AEM.00078-21-s0001.pdf]

Supplemental Material:

Supplemental Methods:

SIP RNA Extraction, Density Fractionation, and qRT-PCR

RNA from the incubator and shipboard SIP experiments was extracted using the mirVana miRNA isolation kit with added bead-beating step using RNA PowerSoil beads (Fortunato and Huber 2016). Extracted RNA (100 µl) was then DNase treated using the Turbo-DNase kit. Gradient preparation, isopycnic centrifugation, and fractionation were performed as described in Fortunato and Huber (2016). Each gradient was fractionated into 12 tubes of approximately 410 µl and the refractive index of each was measured to determine density. RNA was then precipitated using ice-cold isopropanol and washed with 70% ethanol as described in Lueders (2010). RNA concentration of each fraction was determined using the RiboGreen quantification kit and a Gemini XPS plate reader. RT-qPCR was completed using the KAPA SYBR FAST One-Step qRT-PCR kit and universal primers Pro341F and Pro805R (Takahashi et al 2014). Primers were optimized to a final concentration of 0.2 µM and 0.4 µM for the forward and reverse primer respectively. *E. coli* ribosomal RNA was used to construct a standard curve. RT-qPCR results were used to determine the ratio of maximum quantity of 16S rRNA within each set of fractions, that is the ratio of 16S rRNA in a given fraction to the maximum copy number across all fractions.

SIP metatranscriptomic library preparation

Using results from RT-qPCR, four fractions from each of the ^{12}C and ^{13}C samples from the shipboard and incubator samples were sequenced, including fractions with the peak number of 16S rRNA genes and fractions on either side of the peak, for a total of 16 metatranscriptomic libraries. For each SIP metatranscriptomic library, double stranded cDNA was constructed using SuperScript III First-strand synthesis system and mRNA second strand synthesis module. Double stranded cDNA was sheared to a fragment size of 275bp using a Covaris S-series sonicator. SIP metatranscriptomic library construction was completed using the Ovation Ultralow Library DR multiplex system. following manufacturer's instructions. Sequencing was performed on an Illumina NextSeq 500 at the W.M. Keck sequencing facility at the Marine Biological Laboratory. All libraries were paired-end, with a 30 bp overlap, resulting in an average merged read length of 275 bp.

Metagenomic and metatranscriptomic library preparation

The 47mm flat filters were cut in half with a sterile razor, with half used for DNA and half used for RNA extraction. RNA was extracted using the mirVana miRNA isolation kit with added bead-beating step using RNA PowerSoil beads. A total volume of 100 µl was extracted and was then DNase treated using the Turbo-DNase kit (Ambion), purified, and concentrated using the RNAeasy MinElute kit. Ribosomal RNA removal, cDNA synthesis, and metatranscriptomic library preparation was carried out using the Ovation Complete Prokaryotic RNA-Seq DR multiplex system following manufacturer instructions. Prior to library construction, cDNA was sheared to a fragment size of 275 bp using a Covaris S-series sonicator. For DNA extraction, the DNA filter was first rinsed with sterile Phosphate Buffered Saline (PBS) to remove RNAlater and then was extracted using a phenol-chloroform method adapted from Crump et al. (2003) and Zhou et al. (1996). DNA was then sheared to a fragment size of 275 bp using a Covaris S-series sonicator. Metagenomic library construction was completed

using the Ovation Ultralow Library DR multiplex system (Nugen) following manufacturer instructions. Metagenomic and metatranscriptomic sequencing was performed on an Illumina Nextseq 500 at the W.M. Keck sequencing facility at the Marine Biological Laboratory. All libraries were paired-end, with a 30 bp overlap, resulting in an average merged read length of 275 bp.

Analysis of Marker 33 metagenome, metatranscriptome, and RNA-SIP metatranscriptomes:

To identify ribosomal RNA reads, the Marker 33 metatranscriptome and RNA-SIP metatranscriptomes were mapped to SILVA SSU and LSU databases (release 123, Pruesse et al 2007) using Bowtie2 with a local alignment and default settings (v2.0.0-beta5 Langmead and Salzberg 2012). For the Marker 33 metatranscriptome, identified rRNA reads were removed as described in Fortunato et al. (2018). For the RNA-SIP metatranscriptomes, 16S rRNA reads were taxonomically identified via MOTHUR (v1.37, Schloss et al 2009) using the Greengenes 16S rRNA taxonomic database (August 2013 release, McDonald et al 2012).

The Marker 33 metagenome was assembled as described in Fortunato et al. (2018) and contigs were submitted to the DOE Joint Genome Institute's Integrated Microbial Genome Metagenomic Expert Review (IMG/MER) annotation pipeline for Open Reading Frame (ORF) identification and functional and taxonomic annotation (Markowitz et al 2012). Identified ORFs from the Marker 33 metagenome were annotated against the KEGG orthology (KO) database as described in Fortunato et al (2018). Metagenomic reads were mapped to each ORF using Bowtie2 (v2.0.0-beta5 Langmead and Salzberg 2012), with end to end alignment. For the Marker 33 metatranscriptome as well as all 16 RNA-SIP metatranscriptomes, non-rRNA transcripts were mapped to ORFs identified in the Marker 33 metagenome. After mapping, abundances for each ORF were normalized to gene or transcript length. Metagenomic read abundance was normalized to the number of Reads per Kilobase per Genome (RPKG). Number of genomes per metagenome was estimated using hits to the single-copy gene, DNA-directed RNA polymerase beta subunit gene (*rpoB*). The Marker 33 metatranscriptome and all RNA-SIP metatranscriptomes were normalized to the number of Transcripts Per Million reads (TPM). Normalized KO abundances were used for bubble plot construction, hierarchical clustering, and heatmap generation. Hierarchical clustering of samples (average-linkage method) was carried out using the statistical program R (v3.3.2, R-Development-Core-Team 2011) and the R package *pvclust*. Heatmaps were constructed in R using the package *heatmap3*.

To determine taxonomy of non-rRNA transcripts as shown in Figure 3, the RNA-SIP metatranscriptomes were assembled using CLC Genomics Workbench (v 7.0) using default settings and submitted to IMG/MER for ORF identification. The taxonomy for each ORF was determined using the Phylogenetic Distribution tool in IMG, part of the IMG/MER annotation pipeline. To determine taxonomic abundance, the non-rRNA reads were then mapped to each ORF using Bowtie2 (v2.0.0-beta5 Langmead and Salzberg 2012).

References:

Crump BC, Kling GW, Bahr M, Hobbie JE (2003). Bacterioplankton community shifts in an arctic lake correlate with seasonal changes in organic matter source. *Appl Environ Microb* **69:** 2253-2268.

Fortunato CS, Huber JA (2016). Coupled RNA-SIP and metatranscriptomics of active chemolithoautotrophic communities at a deep-sea hydrothermal vent. *Isme J* **10:** 1925-1938

Fortunato CS, Larson B, Butterfield DA, Huber JA (2018). Spatially distinct, temporally stable microbial populations mediate biogeochemical cycling at and below the seafloor in hydrothermal vent fluids. *Environ Microbiol* **20:** 769-784.

Lueders T (2010). Stable Isotope Probing of Hydrocarbon-Degraders. In: Timmis K (ed). *Handbook of Hydrocarbon and Lipid Microbiology*. Springer Berlin Heidelberg. pp 4011-4026.

Langmead B, Salzberg SL (2012). Fast gapped-read alignment with Bowtie 2. *Nat Meth* **9:** 357-359.

Markowitz VM, Chen IMA, Chu K, Szeto E, Palaniappan K, Grechkin Y *et al* (2012). IMG/M: the integrated metagenome data management and comparative analysis system. *Nucleic Acids Research* **40:** D123-D129.

McDonald D, Price MN, Goodrich J, Nawrocki EP, DeSantis TZ, Probst A *et al* (2012). An improved Greengenes taxonomy with explicit ranks for ecological and evolutionary analyses of bacteria and archaea. *Isme J* **6:** 610-618.

Pruesse E, Quast C, Knittel K, Fuchs BM, Ludwig WG, Peplies J *et al* (2007). SILVA: a comprehensive online resource for quality checked and aligned ribosomal RNA sequence data compatible with ARB. *Nucleic Acids Research* **35:** 7188-7196.

R-Development-Core-Team (2011). R: A language and environment for statistical computing. *R Foundation for Statistical Computing*.

Schloss PD, Westcott SL, Ryabin T, Hall JR, Hartmann M, Hollister EB *et al* (2009). Introducing mothur: Open-Source, Platform-Independent, Community-Supported Software for Describing and Comparing Microbial Communities. *Appl Environ Microb* **75:** 7537-7541.

Takahashi S, Tomita J, Nishioka K, Hisada T, Nishijima M (2014). Development of a prokaryotic universal primer for simultaneous analysis of Bacteria and Archaea using next-generation sequencing. *Plos One* **9:** e105592.

Zhou JZ, Bruns MA, Tiedje JM (1996). DNA recovery from soils of diverse composition. *Appl Environ Microb* **62:** 316-322.

Supplemental Tables and Figures:

Table S1: Chemical measurements of diffuse fluid from Marker 33 vent in 2015. Percent seawater was calculated via the conservative tracer magnesium (Mg): $[Mg]_{vent}/[Mg]_{seawater} *100$. Data from Fortunato et al. 2018.

	**Marker 33 2015**
Depth (m)	1516
Temperature (°C)	40.6
Percent Seawater	85.0
pH	5.6
H_2S (µmol/kg)	627.7
SO_4^{-2} (µmol/kg)	22.9
H_2 (µM)	0.1
CH_4 (µmol/kg)	27.6
O_2 (µM)	16.7
Microbial cells per mL	7.2×10^5

Table 2: Statistics for Epsilonbacteraeota metagenome assembled genomes (MAGs). MAGs were selected manually using tetranucleotide frequency and differential coverage. Percent completeness and contamination was calculated in Anvi'o. For comparison these statistics were also calculated using CheckM.

**MAG Name**	**Length (Mb)**	**No. of Contigs**	**GC Content**	**ANVI'O Completeness**	**ANVI'O Contamination**	**CheckM Completeness**	**CheckM Contamination**
Axial_Epsilon_Bin1	0.43	289	28.7	16.1	0.0	24.5	0.0
Axial_Epsilon_Bin2	0.37	237	29.3	13.8	0.9	22.6	1.7
Axial_Epsilon_Bin3	1.75	397	47.9	93.8	4.0	96.5	2.1
Axial_Epsilon_Bin4	0.78	226	50.2	52.0	3.3	53.7	1.7
Axial_Epsilon_Bin5	0.62	190	48.7	38.9	2.4	35.9	0.6
Axial_Epsilon_Bin6	0.22	170	47.2	18.7	1.7	20.1	0.7
Axial_Epsilon_Bin7	0.31	179	30.9	13.3	0.0	21.2	0.0
Axial_Epsilon_Bin8	0.27	168	29.6	14.0	0.2	20.0	0.0
Axial_Epsilon_Bin9	0.23	145	28.4	22.2	0.5	21.0	0.3
Axial_Epsilon_Bin10	1.06	367	50.1	75.2	4.1	70.2	4.3

Table S3: Results of temperature distribution testing. The experiment was conducted in an ice bath in the lab to determine the steady state temperature distribution throughout the incubation bag relative to the temperature measured at the controlling RTD sensor used in the field deployments. Sensors were placed throughout the bag to measure variation both longitudinally and radially. Longitudinal variation was measured by sensors placed at the controlling RTD sensor, middle and distal end of the bag, and held in place with a custom bracket fabricated to fit snugly within the incubation bag. Radial variation was measured using sensors placed in the longitudinal center, and at the top and bottom surface of the bag. Sensors generally all showed good agreement with each other and with the controlling RTD sensor used to monitor temperature during field deployment. We observed a small but constant offset between bag temperature and controlling RTD temperature, but these lab temperature distribution results are expected to be the worst case, with mixing driven solely by natural convection. In the field experiments there will be additional mixing from vehicle motion. Note, thermocouple numbers reference the RTD temperature probe readings and thermistor numbers represent readings from bag sensors.

Incubator Setpoint (°C)	Thermocouple Average (°C)	Thermocouple Std. Dev. (°C)	Thermistor Average (°C)	Thermistor Std. Dev. (°C)	Duration (minutes)
28	28.29	0.04	30.24	0.32	10.7
30	30.36	0.22	32.45	0.32	17.3
52	50.65	0.12	53.99	0.30	5.2
55	55.59	0.04	58.85	0.46	5.7
80	80.32	0.09	84.13	0.67	7.2

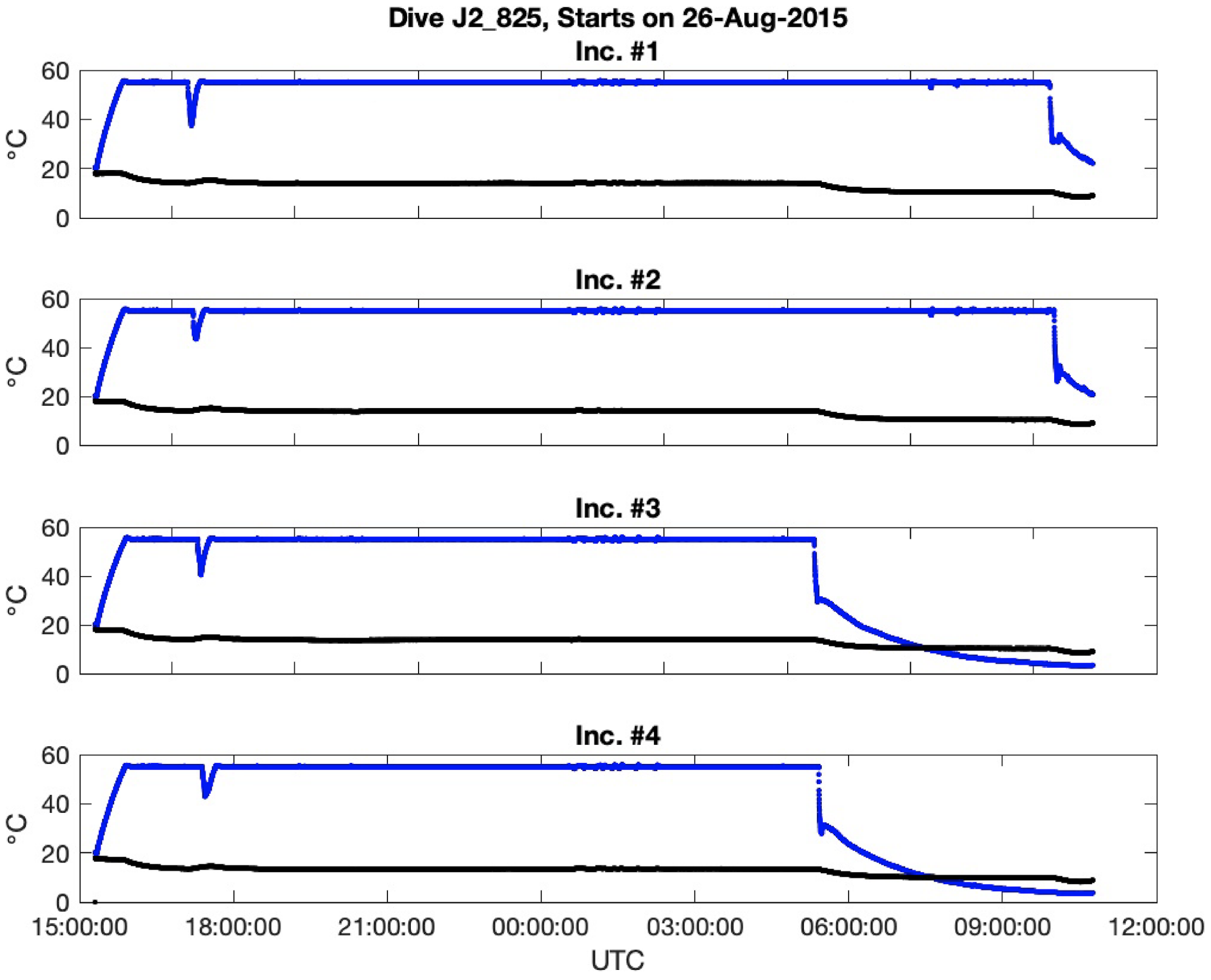

Figure S1. Temperature record of the four incubator units during Jason Dive 825 at Marker 33 with all positions heated to 55°C. Blue line shows measured temperature inside the incubation chamber. The brief dip in measured incubation temperature before 18:00 UTC corresponds to intake of 40°C vent fluid into the incubator, marking the beginning of the incubation. Temperatures were very stable throughout the incubation. The black line is the temperature inside the electronics pressure case.

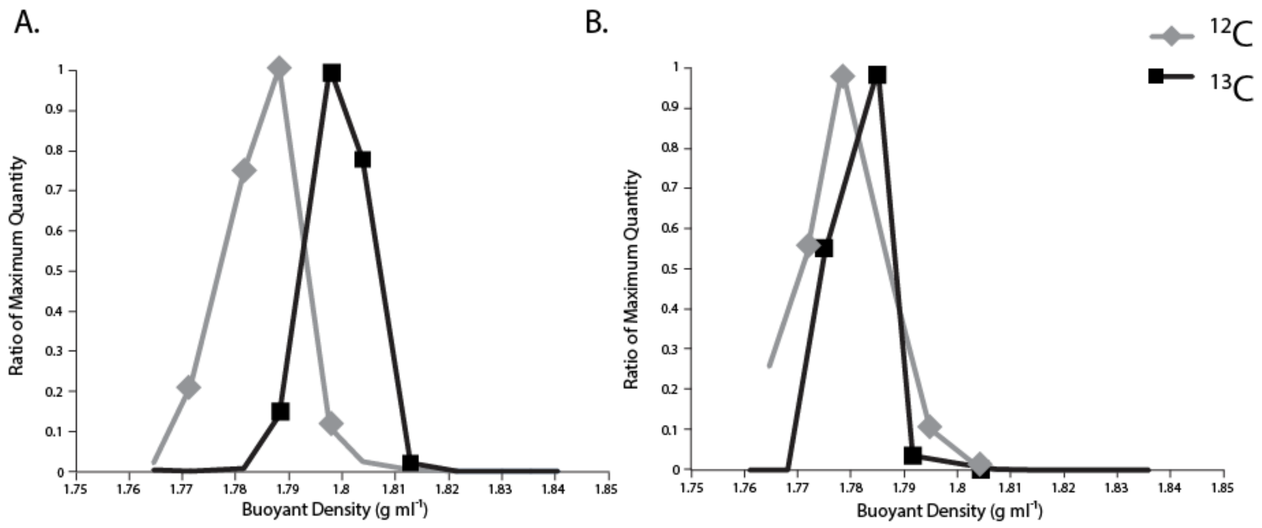

Figure S2: Fractionation plots of (A) Shipboard and (B) Incubator RNA-SIP experiments at 16 hours. Buoyant density (g ml^{-1}) of each fraction is depicted on the x-axis and amount of 16S rRNA as determined by RT-qPCR is on the y-axis. Amount of 16S rRNA is displayed as the ratio of maximum quantity in order to compare between experiments.

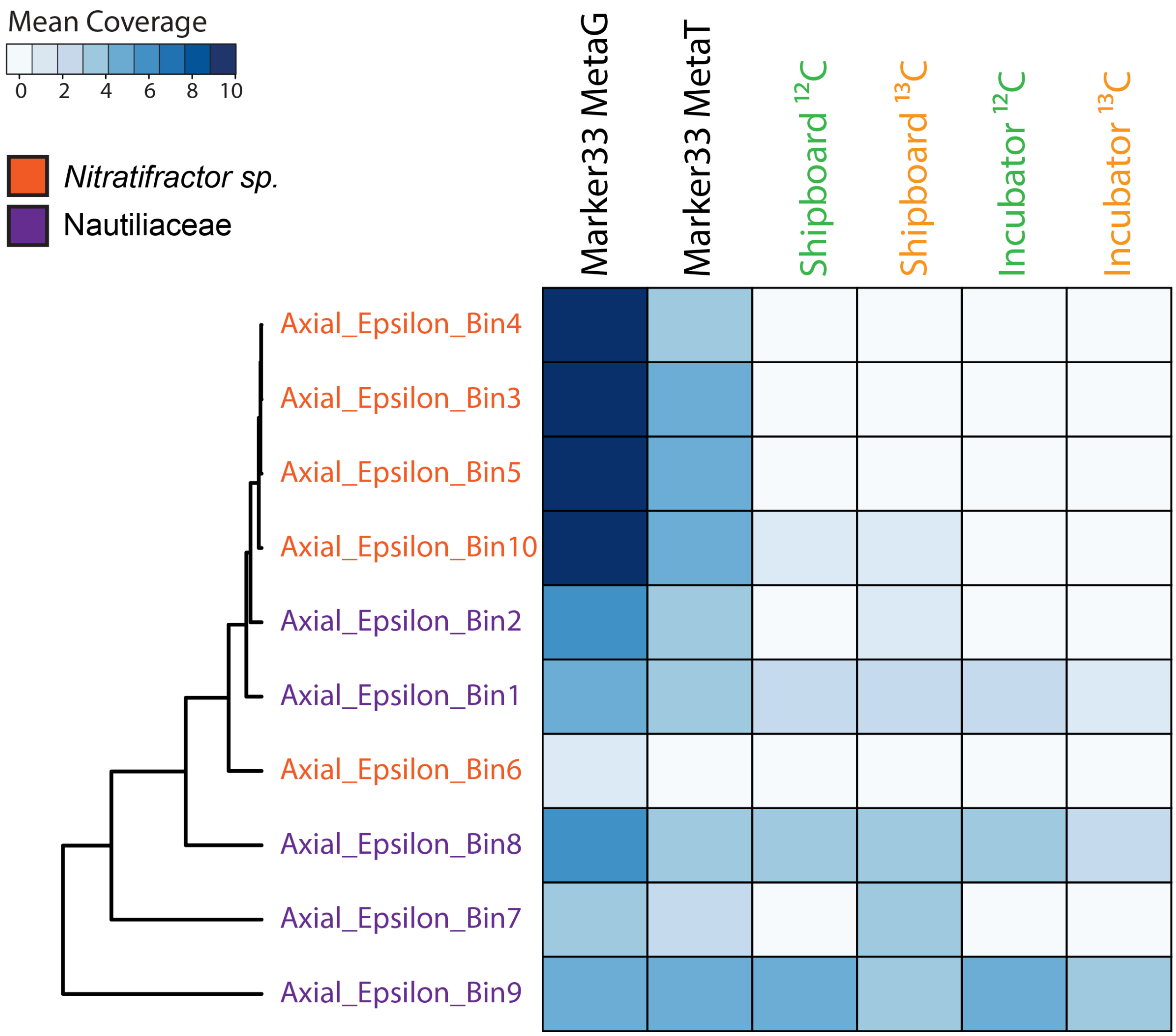

Figure S3: Heatmap of mean coverage across the RNA-SIP experiments of metagenome assembled genomes (MAGs) taxonomically identified as thermophilic Epsilonbacteraeota, specifically either the genus *Nitritifactor* (orange) or the family Nautiliaceae (purple). Coverage of bins within the 2015 Marker33 metagenome and metatranscriptome was included for comparison to SIP fractions. Fractions from each of the four RNA-SIP experiments have been collapsed and mean coverage summed. Scale depicts range of mean coverage across MAGs.

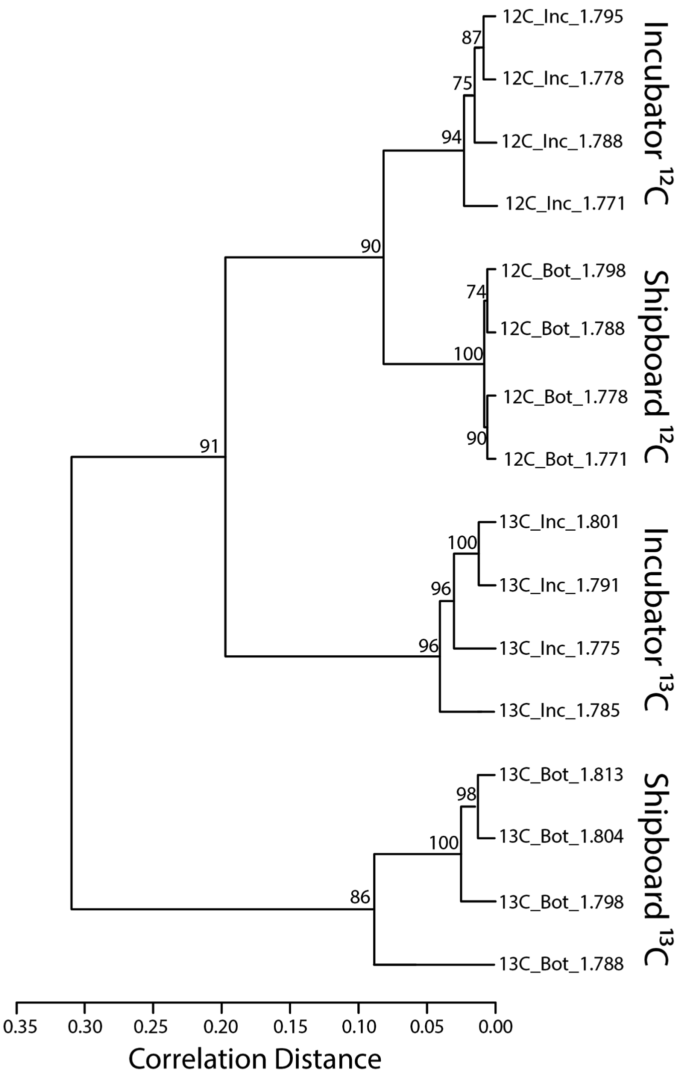

Figure S4: Hierarchical clustering of all shipboard and incubator RNA-SIP experiments based on normalized abundance of annotated (KO) transcripts.

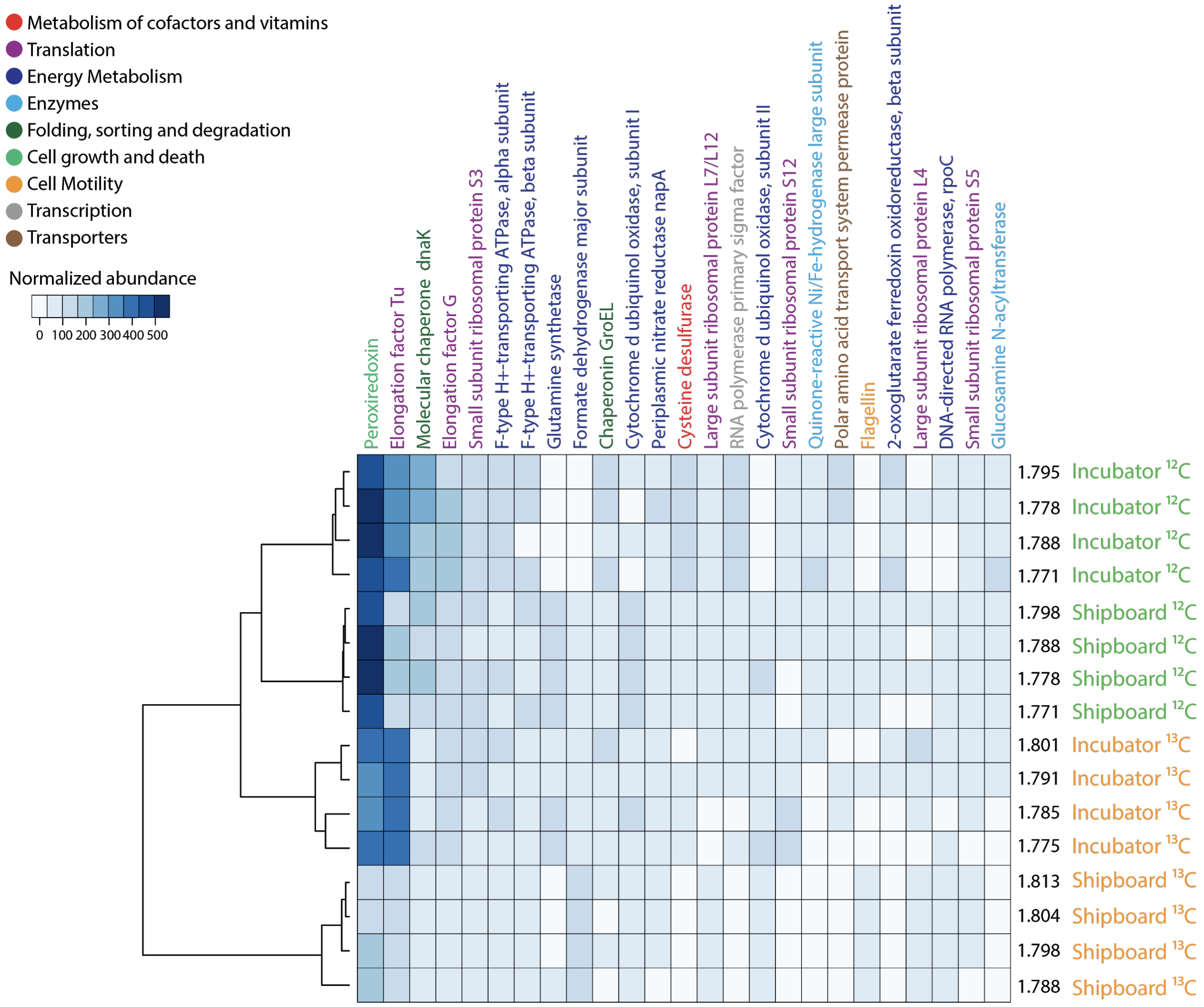

Figure S5: Heatmap depicting the twenty-five most abundant annotated transcripts across all RNA-SIP experiments. Transcripts are colored by the KEGG ontology metabolic group. The dendrogram depicts the degree of similarity among the RNA-SIP fractions based on the normalized abundance of the transcripts. Scale depicts the range of normalized abundance, as determined by Transcripts per Million (TPM).

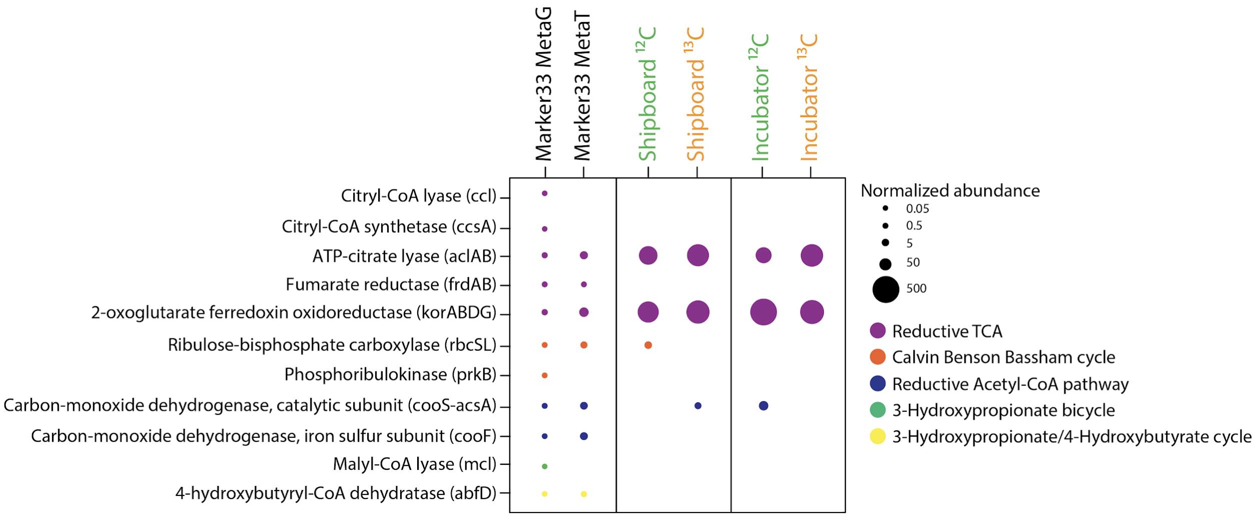

Figure S6: Normalized abundance of annotated genes and transcripts for the five main carbon-fixation pathways identified within the 2015 Marker 33 metagenome, metatranscriptome, and the shipboard and seafloor incubator RNA-SIP experiments. Fractions from each of the four RNA-SIP experiments have been collapsed to reflect the normalized abundance of each gene in the entire experiment

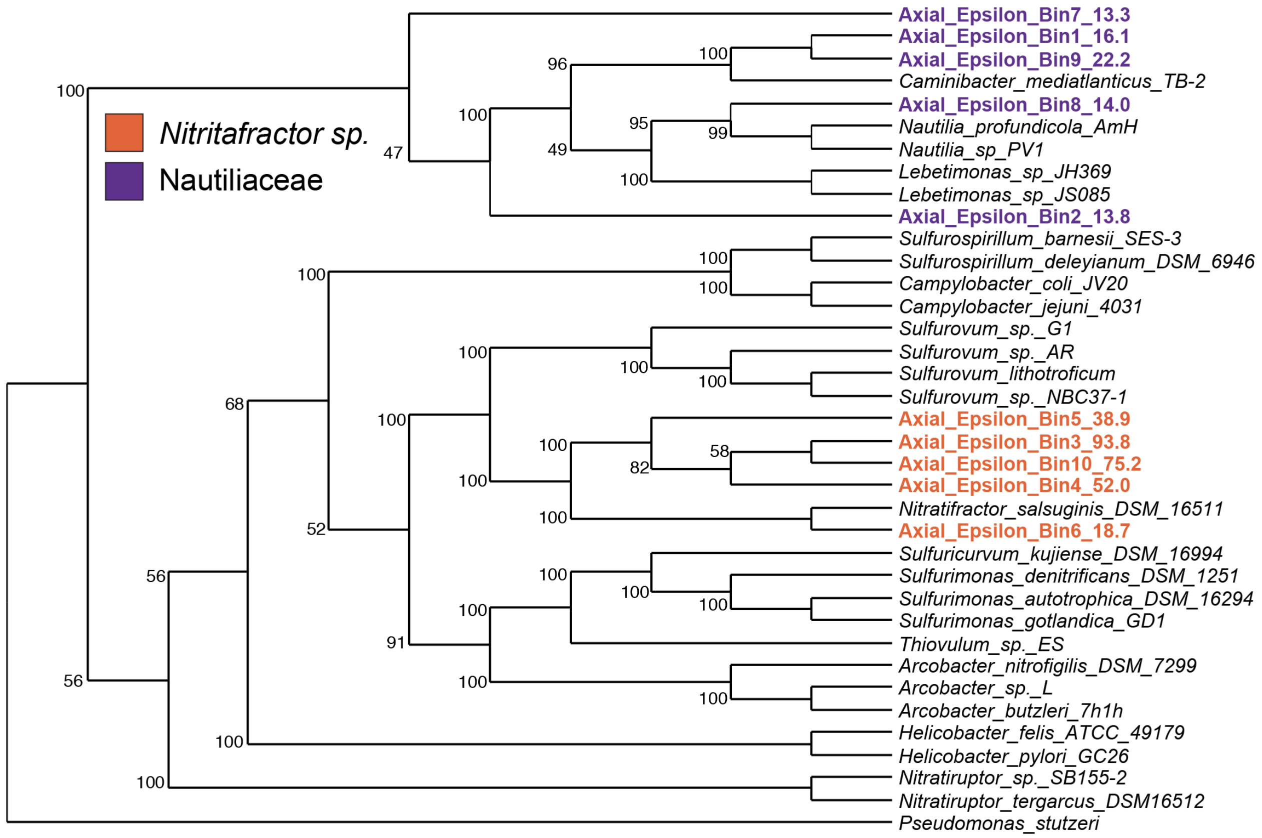

Figure S7: Concatenated marker gene tree of thermophilic Epsilonbacteraeota metagenome assembled genomes (MAGs). Amino acid alignments were constructed using the program Phylosift using a reference set of 37 marker genes. Tree was constructed using RAxML. MAG completeness, as determined via CheckM, is denoted at the end of each MAG name. Legend denotes the assigned taxonomy of MAGs as determined in *Anvi'o*.
